# Parameters Tailoring on the Deposition of Hydroxyapatite by Pulsed Electrical Discharge

**DOI:** 10.3390/ma17184583

**Published:** 2024-09-18

**Authors:** Stefan Alexandru Laptoiu, Mihai Ovidiu Cojocaru, Marian Miculescu, Mihai Branzei

**Affiliations:** 1 Department of Metallic Materials Science and Physical Metallurgy, National University of Science and Technology Politehnica Bucharest, 060042 Bucharest, Romania; stefan.laptoiu@stud.sim.upb.ro (S.A.L.); mihai.branzei@upb.ro (M.B.); 2 Section IX-Materials Science and Engineering, Academia of Technical Science Romania, 010413 Bucharest, Romania

**Keywords:** electrical impulse discharge, hydroxyapatite, biocompatible alloy, bioinert metals, experimental programming, the electrical power of the source, specific surface processing time, relative mass variations, doped bioceramic layer

## Abstract

The creation of strong adhesive layers of hydroxyapatite-based bioceramics (with or without bioinert metals, such as Ta, Ag, and Ti) on biocompatible metallic supports enhances the local biofunctionalization of surfaces. The processing of electroconductive materials using electrical impulse discharges is versatile, enabling precise coating of selected areas with perfectly adherent layers of varying thicknesses. This study aims to quantify the effects of varying the electrical power from the source generating the impulse discharge and the specific processing time per unit area of the cathode (made of titanium alloy) on the relative mass increase of the cathode. The anode comprised a mixture of hydroxyapatite powder and a self-polymerizing electroconductive acrylic resin in a tantalum sheath. The effects of the parameter adjustments on single-layer deposition adherence were quantified using a central composite design to build a second-order orthogonal model. The most significant difference in relative mass was observed with a low-power source (5 W) ensuring the electrical discharge impulse, combined with the longest specified surface treatment time (17.5 s/cm^2^ on a 4 cm^2^ surface) for a single layer presenting the largest mass increase of 0.153% of the original mass. This study aimed to enhance the performance of medical implants by optimizing surface biofunctionalization through robust hydroxyapatite-based bioceramic adhesive layers on metallic supports, determining the optimal electrical power and processing time for cathode mass increase during deposition processes, and analyzing parameter adjustments using second-order statistical orthogonal central composite programming, with a focus on single-layer deposition to identify significant differences in relative mass under specific conditions.

## 1. Introduction

Improving the intrinsic biocompatibility of the active surfaces of metallic implantable medical devices, while maintaining a high level of extrinsic biocompatibility, is a major goal of medical bioengineering [1,2,3]. The main categories of materials from which metallic bioimplants are made depending on their destination are pure metals, such as titanium, tantalum, and gold; iron-based alloys with a high chromium content; stainless steels; alloys based on cobalt with high content of chromium and molybdenum; and alloys with a titanium base. Hydroxyapatite (HA) has emerged as a promising biomaterial with a wide range of applications in reconstructive medicine and biomedical engineering [4,5]. As a major constituent of natural bone, HA exhibits excellent biocompatibility, osteoconductivity, and biodegradability, making it an ideal material for various bone replacement applications, both orthopedic and dental [6]. Ti6Al4V is a titanium alloy with high strength, low density, high fracture toughness, excellent corrosion resistance, and superior biocompatibility [7]. However, Ti6Al4V does not satisfy all the requirements of tribological engineering owing to its disadvantages, such as low surface hardness, high friction coefficient, and poor abrasive wear resistance [8]. There are reports of inflammatory reactions around these implants due to the formation of avascular fibrous tissue encapsulating the implant [9]. Numerous methods have been identified for the deposition of apatite/hydroxyapatite (Ca10(PO4)6(OH)2) on metallic surfaces, including spray techniques (plasma, flame, and magnetron), pulsed laser deposition, electron beam techniques, sol–gel techniques, dip coatings, electrochemical and electrophoretic deposition, biomimetic, hydrothermal, and micro-arc oxidation [10,11,12,13,14,15]. The deposition technique using electrical pulse discharges [16] could represent an attractive technological alternative if hydroxyapatite becomes electroconductive [17].

Electrical impulse discharges, also known as electrical spark deposition (ESD), discharge the energy stored in a capacitor bank and generate a plasma arc for a short period (1–10 s) between the electrode (anode) and substrate (cathode). The generated arc can vaporize the electrode, which is subsequently accelerated by the electric field towards the substrate, resulting in a coating with strong adhesion [18]. Physically, the voltage regulates the arc range between the electrode and substrate, while the capacitors determine the arc duration [19]. The most prominent advantage of ESD is its ability to easily select the deposition area and control the thickness of the deposited layer [20]. Another benefit of this method is that energy is emitted in pulses, which minimizes the temperature in the deposition area. This reduces the occurrence of distortions and residual stress in the product [21]. However, ESD also has some disadvantages, particularly an increased likelihood of producing a rather amorphous structure, which can lead to significant roughness, microcracks, and thinning of deposited layers [22]. Metals, alloys, and composites coated using the ESD electrospark deposition process include stainless steel, Ti-6Al-4V, and Ni-based superalloys [23]. The deposition of ceramics has been demonstrated in studies such as [24] to prepare cermet coatings based on Mo2FeB2 in Ar and air.

ESD is used to repair critical components manufactured from high-cost materials such as parts used in nuclear reactors [25] and the aeronautical [26] and naval industries [27]. This study aimed to enhance the performance of medical implants by optimizing surface biofunctionalization through robust hydroxyapatite-based bioceramic adhesive layers on metallic supports, determining the optimal electrical power and processing time for cathode mass increases during deposition processes, and analyzing parameter adjustments using second-order statistical orthogonal central composite programming, with a focus on single-layer deposition to identify significant differences in relative mass under specific conditions.

## 2. Work Methodology, Materials, and Equipment Used in the Research

Experimental research aims to quantify the effects of variations in some technological parameters of interest in the deposition process through electrical impulse discharges, specifically for depositing an initially electrically nonconductive component, hydroxyapatite, on metallic supports commonly used in medical implants. This involves using an experimental design, the active experiment method, initially using a version of second-order orthogonal compositional central programming [28].

The explanation of the regression equations, which express the effects of variations in independent parameters on the dependent variables of interest, involved the following mandatory stages (after adopting a specific type of experimental design):

- Establishment of independent parameters, ranges, and limits of variation.
- Establishment of second-order orthogonal compositional central programming (concrete experimental conditions).
- Determination of the coefficients of the regression equation.
- Determination of the dispersion of the reproducibility of the experiment (a minimum of three experiments were performed under identical conditions).
- Statistical verification of the coefficients of the nonlinear model.
- Verification of the hypothesis regarding concordance of the adopted nonlinear model.

In this study, we used electrical impulse discharges to improve the top layer of the titanium alloy Ti6Al4V, with hydroxyapatite powder (Ca10(PO4)6(OH)2) obtained from bovine bone that had been ground using a ball mill to obtain a particulate dimension of 10 μm. To ensure the electroconductivity of the hydroxyapatite, it was mixed in equal proportions with an electroconductive resin powder from Metkon DMT CON (Metkon Instruments Inc., Bursa, Turkey) and mixed with Duracril plus from spofadental as a binder. The mixture was injected using tantalum foil rolled into a tube. The deposition was performed using an MPI-702ER device manufactured in the Moldavian Republic. The device had four energy settings (A1, A2, B1, and B2) and a potentiometer to adjust the rotation velocity. In the first step, to determine the device parameters, we assigned the power per pulse for each energy setting. Measurements were made between the electrode and anode using a RIGOL DS1062CA digital oscilloscope, while the amperage was measured with a multimeter across a 2Ω resistor. The discharge of the capacitor bank produced rectangular pulses.

**Figure 1:**
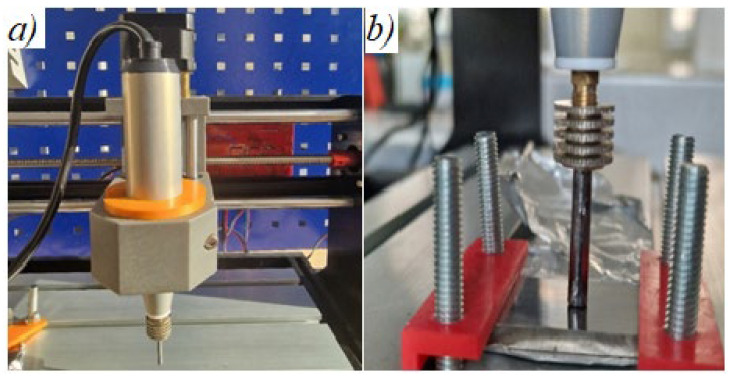
ESD incorporation mode with CNC equipment (**a**) of the electrode (**b**) of the anode.

To maintain consistency in the deposition conditions, we utilized a CNC 3018 Pro computer numerical control system with a working surface measuring 300 × 180 × 40 mm. The axis was equipped with Nema 17 brushless motors (Figure 1). Woodpecker control was performed using LaserGRBL software (https://lasergrbl.com/, accessed on 15 September 2024) to create a 20 × 20 mm square field with lines that filled the space in a vertical and horizontal pattern.

The mass difference was calculated by weighing the titanium in the test probes before and after the deposition using the analytical balance Kern ALJ (KERN & SOHN GmbH, Ebingen, Germany). For analysis of the results, we used an optical microscope (Zeiss Z1m Observer microscope, Axio Vision 4.8/038-12837, (ZEISS AG, Oberkochen, Germany) electron microscopy (TESCAN VEGA XMU 8 microscope (TESCAN, Brno, Czech Republic), Philips XL30 ESEM TMP microscope (FEI Company, Hillsboro, OR, USA)), scanning electron microscopy (SEM) using the Phenom ProXSEM (Thermo Fisher Scientific, Waltham, MA, USA) and X-ray diffraction (X-ray Diffractometer, D8 ADVANCE type (Bruker Corporation, Billerica, MA, USA), Cu-K-alpha radiation 1.54056 nm).

## 3. Results, Interpretations

The independent parameters analyzed were *X*_1_—representing the power source—and *X*_2_—the velocity of the anode movement—which provides useful information related to the specific processing time of the surface.

Note that *X*_1_ and *X*_2_ represent the coded expressions of these two independent parameters, and their values vary within the range [−1 to +1]. As a dependent variable, *Y* (natural value), the relative mass variation of the cathode $(\left(\Delta m/m0\times 100\%\right)$ after applying a monolayer of adherence) was adopted. Following the previously presented algorithm, the variation limits of the adopted independent parameters are established in the first stage (Table 1).

Between the coded value of the independent parameters (*X_i_*) and their natural value (*Z_i_*), there is a connection (Equation 1) that allows their quick conversion into natural values:

(1) $$X_{i}=\frac{Zi-Z_{i0}}{\Delta Zi}$$

The significance of the measurements *Z_i_*, *Z_i_*_0_, and Δ*Z_i_* can be found in Table 1.

The experimental design matrix, containing the concrete conditions under which the N = 2^k^ + 2k + 1 = 9 experiments are dictated by the adopted programming variation must be conducted (where k represents the number of independent variables), and the results obtained from the experiment cycle are presented in Table 2.

The statistical processing of the data related to the relative mass variation of the cathode as a result of alloying through experimentally obtained electrical impulse discharges allowed the calculation of the coefficients of the regression equation of the nonlinear model *b*_0_′; *b*_0_; *b_i_*; *b_ij_*; *b_ii_* (Equation 2).

(2) $$b_{0}^{\prime }=\frac{\sum_{u=1}^{N}x_{0u}y_{u}}{\sum_{u=1}^{N}x_{0u}^{2}};\;bi=\frac{\sum_{u=1}^{N}x_{iu}y_{u}}{\sum_{u=1}^{N}x_{iu}^{2}};b_{ij}=\frac{\sum_{u=1}^{N}x_{iu}x_{ju}y_{u}}{\sum_{u=1}^{N}\left(x_{iu}x_{ju}\right)};b_{ii}=\frac{\sum_{u=1}^{N}x_{iu}^{\prime }y_{u}}{\sum_{u=1}^{N}\left(x_{iu}^{\prime }\right)\left(x_{iu}^{\prime }\right)}$$

The calculated regression equation coefficients were validated by comparing their absolute values with the corresponding confidence intervals ($\left|\Delta b\right|$), which were considered statistically significant if their absolute values were greater than those corresponding to their confidence intervals (Equation (3) and Table 3).

(3) $$\left|\Delta b\right|=t_{\alpha ;N}*\left|Sb\right|$$

where $t_{\alpha ;N}$ represents the value of Student’s standard that signifies the threshold α (expresses the probability that the calculated regression equation represents the relationship between the independent and dependent parameters in the analysis), and is the threshold of significance (α), which is set at 0.05, indicating a 95% probability and *N* degrees of freedom (which in this case is equal to the number of experiments).

*N* degrees of freedom (equal to the number of experiments).

*S_b_*—dispersion in the calculation of coefficients (Equation (4)).

(4) $$S_{bo\prime }^{2}=\frac{S_{0}^{2}}{\sum_{u=1}^{N}x_{0u}^{2}}\;;\;\;\;S_{bi}^{2}=\frac{S_{0}^{2}}{\sum_{u=1}^{N}x_{iu}^{2}}\;;\;\;\;S_{bij}^{2}=\frac{S_{0}^{2}}{\sum_{u=1}^{N}\left(x_{iu}x_{ju}\right){↑}^{2}}\;;\;\;\;S_{bii}^{2}=\frac{S_{0}^{2}}{\sum_{u=1}^{N}\left(x_{iu}^{\prime }\right){↑}^{2}}$$

where $S_{bo\prime }^{2}$ represents the dispersion of the reproducibility of the experiment (Table 4).

The estimation of the dispersions in the calculation of the coefficients ($S_{b0\prime }^{2}$; $S_{bo}^{2}$; $S_{bi}^{2}$ $S_{bij}^{2}$; $S_{bii}^{2}$) and the confidence intervals related to the calculated coefficients of the regression equation led to the following conclusions:

The particular form of the regression equation becomes

(5) $$\tilde{Y}=0.043-0.00883X_{1}-0.0495X_{2}+0.0207X_{1}X_{2}+0.0138\left(X_{1}^{2}-2/3\right)$$

which, after processing, takes the form

(6) $$Y=0.0338+0.0138X_{1}^{2}-0.00883X_{1}-0.0495X_{2}+0.0208X_{1}X_{2}$$

Note that *X*_1_ and *X*_2_ represent the coded forms of the independent parameters *z*_1_ and *z*_2_, respectively, and *Y* is the natural value of the dependent parameter under analysis.

This phase validates the decision to adopt the second-order orthogonal compositional central programming as the correct experimental programming method after checking the dispersion caused by the calculated regression equation (Equation (6)) Table 5. $S_{conc}^{2}$ and comparing the calculated Fisher criterion value *F_calc_* (Equation (7)) with a tabled value, *F_tab_*.

(7) $$F_{calc}=\frac{S_{conc}^{2}}{S_{0}^{2}}$$

The results were as follows:

(8) $$F_{calc}=\frac{4.17\;*\;10^{-4}}{7.23\;*\;10^{-5}}=5.76$$

And *F_tab_*_;α;ϑ1;ϑ2_ = *F_tab._*_0.__05;4;2_ = 19.25 > *F_cal_*, where ϑ_1_ = *N*–*K*′ represents the number of degrees of freedom with which the dispersion caused by the regression equation is calculated, equal to the number of experiments (*N*) from which the number of statistically determined coefficients, including the coefficient of the free term, is subtracted; ϑ_2_ = n_0_−1, the number of degrees of freedom with which the dispersion of the reproducibility of the experiment was calculated, $S_{0}^{2}$;*n*_0_ is the number of parallel experiments (performed under identical conditions); *n*_0_ = 3.

**Figure 2:**
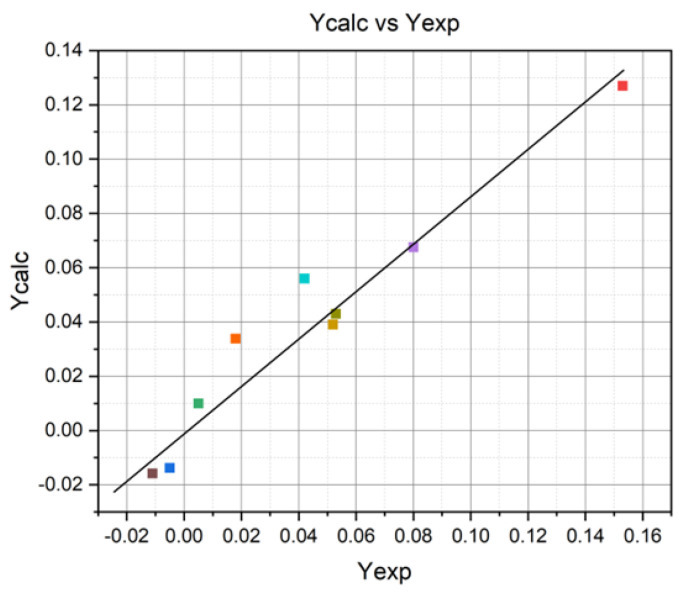
Design–Expert plot of calculated vs. experimental values.

**Figure 3:**
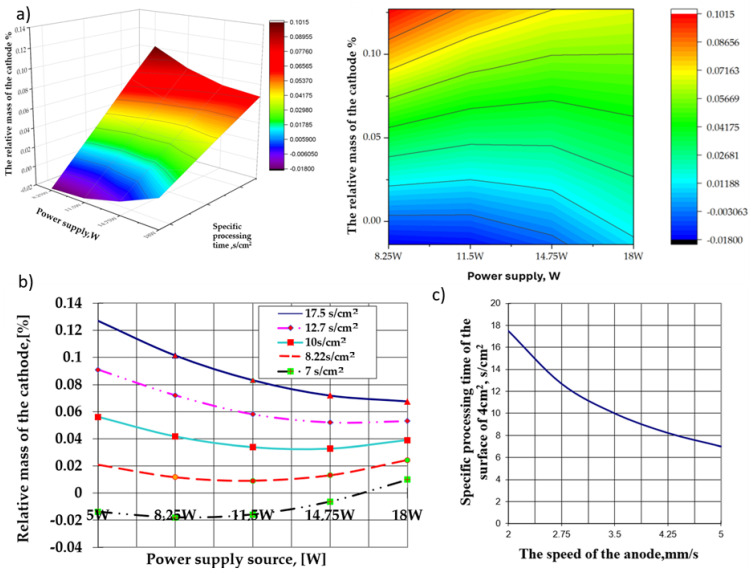
(**a**) The response surface of the regression equation (Equation (6)); (**b**) cross-sections of the response surface at varying values of the specific surface processing time; (**c**) correlation between the specific surface processing time (4 cm^2^—a single pass required to create an adhesion layer) and the speed of the anode.

The model’s precision can be evaluated utilizing a diagnostic diagram of calculated versus experimental values [29]. Figure 2 illustrates the plot of predicted versus experimental values. All experimental data points are situated in proximity to the straight line, indicating the consistency of a normal distribution. A strong correlation between the calculated and experimental values for the response function substantiates the adequacy of the proposed model. An analysis of the calculated and tabulated values of the Fisher criterion highlights that the choice to adopt second-order orthogonal compositional central programming as the method of designing the experiment was correct, and the equation of the calculated model expresses, with maximum probability, the dependence between the two groups of parameters (independent and dependent, respectively). A graphical representation of this equation is presented in Figure 3. 

The analysis of the graphical expressions in Figure 3b reveals several key elements of interest in deposition through electrical impulse discharges. These findings are specific to the first few layers of hydroxyapatite deposited on the surface of the cathode; however, their validity may vary depending on the nature of the support and deposition process.

- A high anode traversal velocity over the cathode surface, associated with the lower power values of the source (5 W in this group of experiments), makes it possible to tear off the particles from the surface of the cathode in the interelectrode plasma cloud, resulting in relative mass loss.
- Observation: The power of the source was considerably lower than the energy released during the electrical impulse discharge in the interelectrode plasma cloud.
- The most significant relative mass increase of the cathode corresponds to the lowest power of the power source (5 W in this case) associated with the longest specific processing time of the cathode (the lowest anode traversal velocity over the cathode surface), which increases the probability of redeposition of any detachment from the cathode surface along with the polar transfer and deposition of the products resulting from anodic erosion.
- An increase in the power of the electrical energy supply source affects the relative mass variation of the cathode, which is strictly dependent on the specific time of its surface processing (the velocity of the anode movement on the cathode surface).
- The decrease in the specific time for processing the cathode surface within the limits of 17.5 ÷ 7.0 s/cm^2^ associated with variations in the power of the energy supply within the limits of 5.0 ÷ 11.5 W implies a decrease in the relative mass of the cathode, which grows more significant as the specific processing time increases.
- When the power of 11.5 W of the supply source is exceeded, the effect is much more attenuated and occurs at specific processing times below 10 s/cm^2^, even when the relative mass variations of the cathode are increased. The effects are related to, and depend on, the ratio between the rate of particle removal from the cathode’s surface and the rate of deposition on its surface. Regardless of the situation, the most substantial relative mass increase recorded by the cathode was obtained at the reduced values of the power of the electrical energy supply source (at the lower limit of the tested range) and the highest value of the specific processing time.

**Figure 4:**
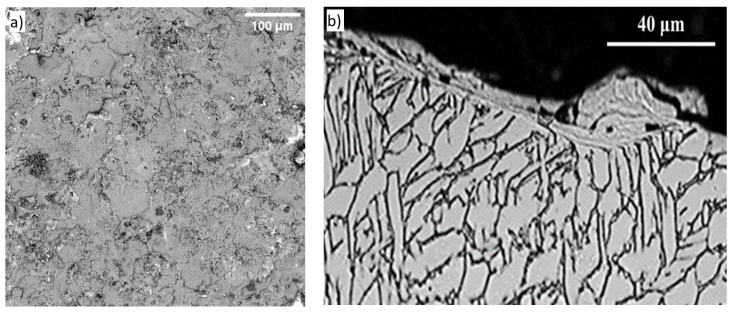
(**a**) SEM analysis of the surface of the hydroxyapatite layer with 500× magnification; (**b**) SEM metallography analysis of the layer.

The SEM surface analysis presented in Figure 4a indicates the presence of pores with sizes ranging from 0.300 to 5 µm and microcracks measuring between 1.430 and 8.280 µm. After one pass, a biocompatible Ti6Al4V alloy layer with a thickness of 50–60 μm was obtained. These features were measured using ImageJ 1.54i software. The topography of the surface was analyzed using scanning electron microscopy, confirming the migration mechanism of the erosion process, as described in [25]. According to this mechanism, the current does not flow uniformly across the entire area of interaction between the electrodes and the plasma jet; instead, it is concentrated in strictly limited portions at any given moment. This results in the formation of interaction zones known as electrode spots, which can be detected using metallographic methods.

**Figure 5:**
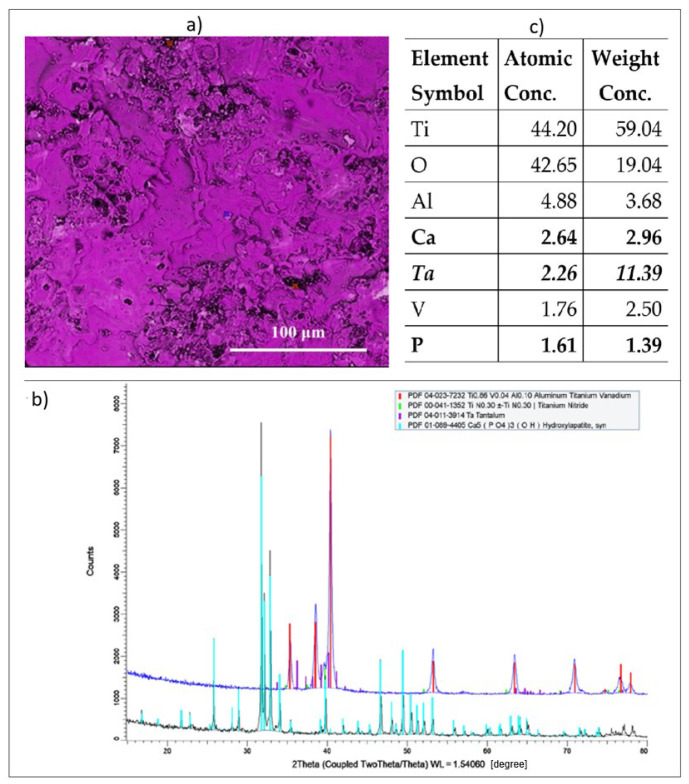
(**a**) analyzed area image of the sample surface, (**b**) EDAX elemental microanalysis spectra on the sample surface, (**c**) EDAX quantitative elemental results.

Analysis utilizing the EDAX method (Figure 5) revealed that the titanium alloy comprised approximately 2.96% calcium, 1.39% phosphorus (highlighted in Figure 5c), and 11.39% tantalum (Figure 5c—added from the electrode material) in combination with the elements of the titanium alloy. The experiments were conducted under the following conditions: (one layer—the adhesion layer; processed surface 4.0 cm^2^ *p* = 5 W). 

## 4. Conclusions

The formation of hydroxyapatite deposits on titanium-based alloy implants using the impulse electric discharge method is feasible with the appropriate preparation of hydroxyapatite, which is achieved by ensuring electroconductivity through mixing with a self-polymerizing electroconductive acrylic component. By developing a regression equation based on a cycle of experience and the adopted programming method, we can precisely determine the best conditions for processing within a given interval, including the power of the energy supply source and specific processing. The successful deposition of a single adhesion layer is essential for obtaining a substantial increase in the cathode mass. Notably, the highest relative mass increase in the cathode 0.153 over the initial results was observed at relatively low power levels of the electrical energy supply source of 5 W combined with extended processing times of 2 mm/s. X-ray diffraction and electron microscopy–elemental chemical microanalysis confirmed the presence of hydroxyapatite in the deposited layer and identified titanium nitride, which was formed owing to the presence of nitrogen ions in the interelectrode plasma cloud. This study focused only on the potential of the impulse electric discharge method for the effective deposition of hydroxyapatite on titanium-based implants. This technique can be used to repair or improve the properties of implants in the surgical field. In the future, it will be necessary to test the mechanical and biointegration of the hydroxyapatite obtained with this method.

## Figures and Tables

**Table 1:** Establishing the base levels and the variation intervals corresponding to the two independent parameters taken into analysis.

Factors	Z_1_-Power of the Feeding Source, W	The Z_2_-Moving Velocity of the Anode, mm/s
Code	*X*_1_	*X*_2_
Base Level, *Z_i_*_0_	11.5	3.5
Level of Variation, Δ*z_i_*	6.5	1.5
Superior Level (+1); *Z_i_*_0_ + Δ*z_i_*	18	5 (7 s/cm^2^)
Inferior Level (−1); *Z_i_*_0_ − Δ*z_i_*	5	2 (17.5 s/cm^2^)

The independent parameter limitations are being given by the superior and inferior limits of the devices. The surface area of the coating was 2 × 2 cm, totaling 4 cm^2^ in Section 2, and the coating was made through a single pass (a single layer of adhesion). In this way, the specific processing time of the cathode surface unit was varied within the limits of 17.5 ÷ 7 s/cm^2^ for the variation of the anode displacement velocity within the limits of 2 ÷ 5 mm/s.

**Table 2:** The matrix of the second-order orthogonal compositional central programming, for k = 2.

Experiment Number	*X*_0_	*X*_1_	*X*_2_	*X*_1_ *X*_2_	$X_{1}\prime ={X_{1}}^{2}-2/3$	$X_{2}\prime =X^{2}-2/3$	$Y=\frac{\Delta m}{m_{0}}\times 100\%$
1	+1	−1	−1	+1	+1/3	+1/3	0.153
2	+1	−1	+1	−1	+1/3	+1/3	−0.005
3	+1	+1	+1	+1	+1/3	+1/3	0.005
4	+1	+1	−1	−1	+1/3	+1/3	0.08
5	+1	+1	0	0	+1/3	−2/3	0.052
6	+1	−1	0	0	+1/3	−2/3	0.042
7	+1	0	+1	0	−2/3	+1/3	−0.011
8	+1	0	−1	0	−2/3	+1/3	0.053
9	+1	0	0	0	−2/3	−2/3	0.018

**Table 3:** Statistical verification of the calculated coefficients of the regression equation.

$S_{b}^{2}$	$\left|S_{b}\right|$	$\left|\Delta_{b}\right|$	$b_{calc}$	Conclusion
$S_{b0\prime }^{2}$ = 8.0377 · 10^−6^	0.00283	-	*b*_0_ = 0.043; *b*_1_ = −0.00883; *b*_2_ = −0.00495; *b*_12_ = 0.0207; *b*_11_ = 0.0138; *b*_22_ = 0.0085	-
$S_{bo}^{2}$ = 2.411 · 10^−5^	0.00491	0.01100		*b*_0_-OK
$S_{bi}^{2}$ = 1.205 · 10^−5^	0.00347	0.00784		*b*_1_;*b*_2_-OK
$S_{bij}^{2}$ = 1.808 · 10^−5^	0.00425	0.00960		*b*_12_-OK
$S_{bii}^{2}$ = 3.617 · 10^−5^	0.00601	0.01360		*b*_11_-OK

The value of Student’s criterion (tabulated value) is 2.26 for a significance threshold α = 0.005 and *N* = 9, and $S_{b0}^{2}\neq S_{b0\prime }^{2}$ $S_{b0}^{2}=S_{b0\prime }^{2}+\left({\bar{x}}_{i}^{2}\right)S_{bii}^{2}$ = $S_{b0\prime }^{2}$ + $\left({\bar{x}}_{i}^{2}\right)S_{bii}^{2}$.

**Table 4:** The calculation of reproducibility of dispersion $S_{bo\prime }^{2}$.

Expression Number	*Yu*	$\Delta Y=\left|Yu-\overset{=}{Y}\right|$	$\Delta \left(Y^{2}\right)$	$\vartheta_{2}$
1	0.018	3.4 · 10^−3^	1.156 · 10^−5^	ϑ_2_ = 3 − 1 = 2
2	0.005	9.6 · 10^−3^	9.216 · 10^−5^	
3	0.021	6.4 · 10^−3^	4.096 · 10^−5^	
	$\overset{=}{Y}$ = 0.0146		$\Sigma \Delta (Y^{2})\;=\;14.468\;*\;10^{-5}$	$S_{0}^{2}$ = 7.234 · 10^−5^

**Table 5:** Calculation of the dispersion caused by the regression equation $S_{conc}^{2}$.

Number	*Y_exp_*	*Y_calc_*	$\Delta Y2={\left|Y_{exp}-Y_{calc}\right|}^{2}$	$\vartheta_{1}=N-K\prime$
1	+0.153	+0.127	6.76 · 10^−4^	$\vartheta_{1}$ = 9 − 5 = 4
2	−0.005	−0.0138	7.74 · 10^−5^	
3	+0.005	+0.0100	2.5 · 10^−5^	
4	+0.08	+0.0675	1.56 · 10^−4^	
5	+0.052	+0.039	1.69 · 10^−4^	
6	+0.042	+0.056	1.96 · 10^−4^	
7	−0.011	−0.0158	2.304 · 10^−5^	
8	+0.053	+0.043	1 · 10^−4^	
9	+0.018	+0.0338	2.49 · 10^−4^	
			ΣΔ*Y*^2^ = 16.7 · 10^−4^	
$S_{\mathrm{conc}}^{2}\;=\;\frac{16.7\;*\;10^{-4}}{4}\;=\;4.17\;*\;10^{-4}$				

## Data Availability

The original contributions presented in the study are included in the article, further inquiries can be directed to the corresponding author.
